# Multi-Walled Carbon Nanotube-Assisted Encapsulation Approach for Stable Perovskite Solar Cells

**DOI:** 10.3390/molecules26165060

**Published:** 2021-08-20

**Authors:** Jin-Myung Choi, Hiroki Suko, Kyusun Kim, Jiye Han, Sangsu Lee, Yutaka Matsuo, Shigeo Maruyama, Il Jeon, Hirofumi Daiguji

**Affiliations:** 1 Department of Chemistry Education, Graduate School of Chemical Materials, Crystal Bank Institute, Pusan National University, Busan 46241, Korea; csj356093@gmail.com (J.-M.C.); ewrb23@gmail.com (K.K.); hanyksw20@naver.com (J.H.); ssang94@naver.com (S.L.); 2 Department of Nano Fusion Technology, Pusan National University, Busan 46241, Korea; 3 Department of Mechanical Engineering, School of Engineering, The University of Tokyo, Tokyo 113-8656, Japan; suko.ut.mech@thml.t.u-tokyo.ac.jp (H.S.); yutaka.matsuo@chem.material.nagoya-u.ac.jp (Y.M.); maruyama@photon.t.u-tokyo.ac.jp (S.M.); 4 Department of Chemical System Engineering, Graduate School of Engineering, Nagoya University, Nagoya 464-8603, Japan

**Keywords:** perovskite solar cells, carbon nanotubes, encapsulation, passivation, packaging, epoxy resin

## Abstract

Perovskite solar cells (PSCs) are regarded as the next-generation thin-film energy harvester, owing to their high performance. However, there is a lack of studies on their encapsulation technology, which is critical for resolving their shortcomings, such as their degradation by oxygen and moisture. It is determined that the moisture intrusion and the heat trapped within the encapsulating cover glass of PSCs influenced the operating stability of the devices. Therefore, we improved the moisture and oxygen barrier ability and heat releasing capability in the passivation of PSCs by adding multi-walled carbon nanotubes to the epoxy resin used for encapsulation. The 0.5 wt% of carbon nanotube-added resin-based encapsulated PSCs exhibited a more stable operation with a ca. 30% efficiency decrease compared to the ca. 63% decrease in the reference devices over one week under continuous operation. Specifically, the short-circuit current density and the fill factor, which are affected by moisture and oxygen-driven degradation, as well as the open-circuit voltage, which is affected by thermal damage, were higher for the multi-walled carbon nanotube-added encapsulated devices than the control devices, after the stability test.

## 1. Introduction

Organohalide perovskite materials have attracted considerable attention, especially in the application of energy harvesting [1,2]. When applied in photovoltaics, these materials yield a high-power conversion efficiency owing to their outstanding properties, namely their wide range of light absorption and long exciton diffusion length [3,4]. Despite the high efficiency of perovskite solar cells (PSCs), insufficient device stability has been the limiting factor in commercialisation. Moreover, the perovskite materials have been reported to degrade easily due to moisture [5], oxygen [6,7], ultra-violet (UV) light, trapped charge [8,9], and heat [10,11,12]. While most researchers have focused on improving the stability of perovskite materials against moisture, it is noteworthy that good packaging technology can prevent water and oxygen intrusion before degradation occurs in the material [13]. The other aforementioned adverse factors can be resolved using UV-cutting glass and charge-transporting materials with a low chemical capacitance [8,9]. However, the thermal damage is difficult to circumvent in such an encapsulated system because the heat is trapped inside the encapsulating glass. As most of the known electronics utilise encapsulation, it is evident that the PSCs will inevitably be encapsulated, which confines the device system. The confinement works favourably in the perspective of moisture and oxygen barriers but unfavourably in heat release. Therefore, it is desirable to develop an encapsulation system that blocks moisture and oxygen as much as possible while releasing heat to the furthest extent. A cover glass is used in any type of encapsulation system, as it is a perfect barrier for both moisture and heat. This implies that the leakage occurs where the UV curable epoxy resin is placed [14,15,16,17]. Thus, the performance of encapsulation is determined by the barrier ability of the epoxy resin. Accordingly, many efforts have been made to develop epoxy resins with better barrier properties [18,19,20].

Since their discovery in 1991 [21], carbon nanotubes have been used in various applications owing to their exceptionally high electrical [22,23] and thermal conductivity [24,25] along the direction of the graphitic tubes. Moreover, carbon nanotubes are highly hydrophobic, with an average tube diameter less than the size of a water molecule, making it difficult for the water molecules to pass through the tubes [26,27]. In addition, the conjugated double bonds present in carbon nanotubes react with radicals, forming strong sigma bonds. This implies that adding carbon nanotubes to epoxy resins can improve the moisture barrier ability and increase thermal conductivity.

Herein, we report the addition of multi-walled carbon nanotubes (MWCNTs or MWNTs) to UV curable epoxy resin sealants used for the encapsulation of inverted-type PSCs, demonstrating improved moisture, oxygen barrier ability, and heat releasing properties. For the standardisation of the experiment, we tested the encapsulation with different structural approaches and applied pressures. The PSCs with MWNT (0.5 wt%)-added encapsulants demonstrated a longer device lifetime than conventional devices. The stability data of the reference devices with no MWNTs showed a faster decrease in the short-circuit current density (*J*_SC_), fill factor (FF), and open-circuit voltage (*V*_OC_) values over time. According to literature and experiments conducted in this work, the decreases in *J*_SC_ and *V*_OC_ originated from moisture intrusion [28,29,30] and thermal damage [12,30,31], respectively. While the degradation caused by moisture and oxygen was more dominant, damage by the trapped heat was insignificant enough to ignore. The addition of MWNTs to the UV epoxy resin alleviated the decreases in *J*_SC_ by ca. 21.5% and FF by ca. 43.5%, which was attributed to the improved moisture and oxygen barrier ability. Furthermore, *V*_OC_ became steady during the entire operation of the solar cells, indicating minimal thermal damage due to the excellent heat-releasing property of the 0.5 wt% MWNT-added UV resin-based encapsulation. The results indicated that incorporating the carbon nanotubes in encapsulation can serve as a gateway to minimising thermal damage and as a barrier to moisture and oxygen attack. Therefore, this study revealed the pathway to stable next-generation thin-film photovoltaic technology, towards the commercialisation of thin-film solar cells.

## 2. Results and Discussion

**Figure 1:**
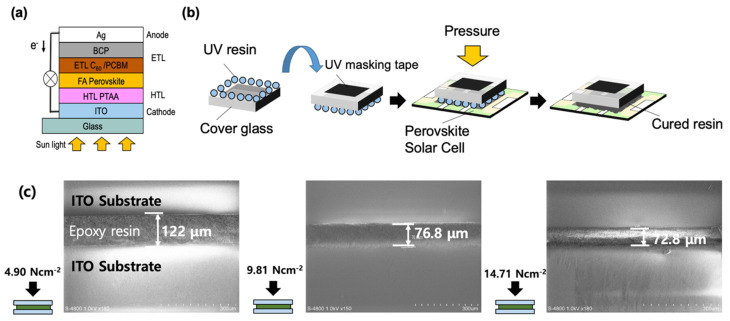
(**a**) Structure of an inverted PSC fabricated in this work; (**b**) packaging process using UV epoxy resin; (**c**) cross-sectional SEM images and thickness of the pressed UV-epoxy resin.

**Figure 2:**
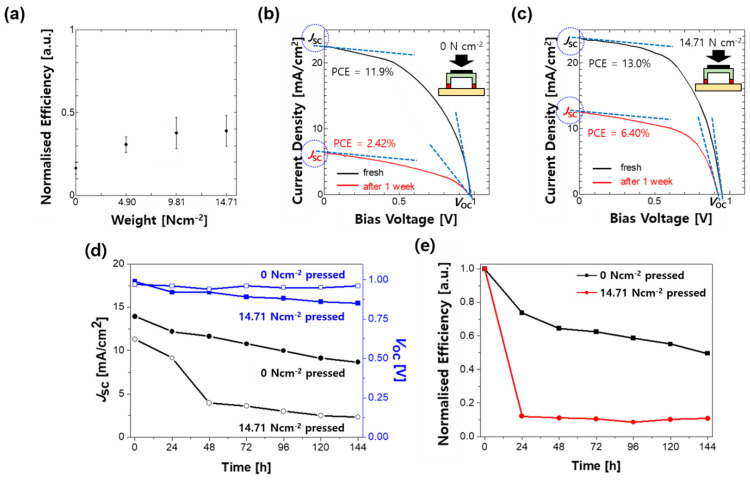
(**a**) Normalised efficiency drop of PSCs encapsulated under different applied pressures during the packaging process left in the atmosphere after one week; (**b**,**c**) *J–V* curves of the encapsulated PSCs under (**b**) 0 N cm^−2^ and (**c**) 14.71 N cm^−2^ before and after being left in the atmosphere for one week; (**d**) changes in *J*_SC_ and *V*_OC_ of the devices encapsulated under the applied pressures of 0 N cm^−2^ and 14.71 N cm^−2^. (**e**) Decrease in the normalised efficiency during one week of the stability test for the PSCs encapsulated under the applied pressures of 0 N cm^−2^ and 14.71 N cm^−2^.

The inverted type PSCs were fabricated in the configuration of indium tin oxide (ITO)/poly(triaryl amine) (PTAA)/perovskite/phenyl-C61-butyric acid methyl ester (PCBM)/C_60_/bathocuproine (BCP)/Ag (Figure 1a). The encapsulation process was standardised to make stability comparisons fair during tests. From our preliminary tests, we discovered that using desiccant, also known as ‘getter’, could reduce the UV light damage on the perovskite film during the curing process, functioning like UV masking tape (Figure S1 in Supplementary Materials). Therefore, we used the getter in all of our encapsulated devices. Based on this, three different structural encapsulation approaches were examined (Figure S2a–d). The cover glass (with and without a cavity) and the applied position of the UV epoxy resin were tested, and a UV curing time of 8 min was used. Different pressures were applied for each structure during the encapsulation (Figure S2e,f). The results showed that the cover glass with a cavity had the most stable operation when UV epoxy resin was applied on the edge, with the UV masking tape protecting the device (Figures S3 and S4). However, the device on which uncured epoxy resin was applied showed the worst device stability, revealing that uncured resin damaged the device (Figure S3c). In addition, all the devices encapsulated under hard pressure exhibited a much higher device stability. It is notable that the applied pressure was the most dominant factor in the device packaging (Figure 1b). Therefore, different specific weight pressures applied during the encapsulation, in particular 0 N cm^−2^, 4.90 N cm^−2^, 9.81 N cm^−2^, and 14.71 N cm^−2^, were tested. The epoxy resin thickness under different pressures was tested by cross-sectional scanning electron microscopy (SEM) (Figure 1c). The thicknesses were 122 μm, 76.8 μm, and 72.8 μm for 4.90 N cm^−2^, 9.81 N cm^−2^, and 14.71 N cm^−2^, respectively. After the UV curing of samples, the device stability of the encapsulated samples was measured. Figure 2a shows that the devices with higher pressure, that is, thinner resin, exhibited greater device stability (Figure S5). Although the 9.81 N cm^−2^ pressure was sufficient to produce device encapsulation with good stability, the optimal pressure was 14.71 N cm^−2^. The current density–voltage (*J–V*) curves of the encapsulated devices, with the applied pressures of 4.90 N cm^−2^ and 14.71 N cm^−2^ before and after one week of durability testing, are shown in Figure 2b and 2c, respectively. From the changes in the *J–V* curves, we can observe that the *J*_SC_ and FF of the devices with an applied pressure of 4.90 N cm^−2^ decreased more significantly than the devices with an applied pressure of 14.71 N cm^−2^ after one week (Figure 2d,e; Table 1). Moreover, the decrease in *J*_SC_ was reportedly linked to degradation by moisture [29]. When water molecules penetrated the epoxy resin and contacted the perovskite photoactive layer, the degradation of the perovskite layer was triggered; NH_2_CHNH_2_PbI_3_ (FAPbI_3_) dissociated to FAI and PbI_2_ by moisture and sunlight [11,30,31,32,33].

NH_2_CHNH_2_PbI_3_ → PbI_2_ + CH_5_IN_2_, (1)

NH_2_CHNH_2_PbI_3_ + H_2_O → NH_2_CHNH_2_PbI_3_H_2_O, (2)

4(NH_2_CHNH_2_PbI_3_) + 2H_2_O → (CH_3_NH_3_)_4_PbI_6_2H_2_O + 3PbI_2_. (3)

Due to such degradation, the light absorption coefficient of the perovskite layer decreased, which in turn decreased *J*_SC_. Additionally, the degradation of the perovskite was reported to increase the contact resistance at the perovskite interfaces between PTAA and PCBM [34]; this was reflected in the increase in the series resistance (*R*_S_) of our devices, which entailed a decrease in FF. Therefore, we can conclude that the moisture penetration through the UV epoxy resin was the main reason for the decrease in the *J*_SC_ and FF. As more moisture penetrates the devices encapsulated with the applied pressure of 4.90 N cm^−2^, more significant decreases in *J*_SC_ and FF were observed than in the devices with the applied pressure of 14.71 N cm^−2^.

The decrease in the open-circuit voltage (*V*_OC_) of the devices was significantly smaller than that of the *J*_SC_ and FF during the durability test. The leakage current at the hole-transporting layer (HTL) and the electron-transporting layer (ETL), causing hole–electron recombination, was reported to reduce *V*_OC_ [35,36]. However, in this case, the *V*_OC_ decrease was not due to the leakage current as the shunt resistance values were almost the same. It was reported that thermal damage to the perovskite reduced *V*_OC_ [28,37,38,39,40]. Furthermore, the encapsulated devices with a stronger pressure of 14.71 N cm^−2^ resulted in a slightly greater *V*_OC_ loss after one week than the devices with a pressure of 0 N cm^−2^. Devices to which a nitrogen gun was constantly blown during the stability test to negate the heat damage, showed no *V*_OC_ loss either (Figure S6). Thus, we can reasonably deduce that the trapped heat caused the reduction in *V*_OC_ as good encapsulation did not only let the air and moisture out but also retained the heat. This implied that it was desirable to have a system that could block moisture and oxygen and release heat more effectively.

**Figure 3:**
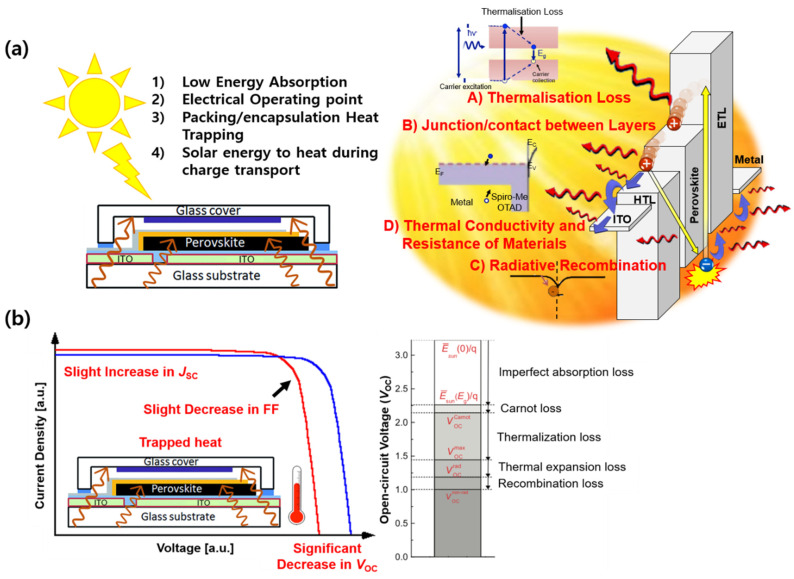
(**a**) Origin of the heat trap categorised by possible sources (**left**) and the detailed illustration of heat generation during the operation of PSC (**right**) (**b**) Mechanism of PSC degradation due to heat trapping during the PSC operation and the breakdown on the origin of the *V*_OC_ loss related to the change in photovoltaic performance of the PSC.

Therefore, the role of the UV epoxy resin is critical in exhibiting such properties. We introduced MWNTs into the UV epoxy resin to realise this. It has been reported that adding MWNTs improves the moisture and oxygen barrier ability and increases the thermal conductivity of the UV epoxy resin [41,42]. Accordingly, we added small amounts of MWNTs to the UV epoxy resin and tested its effect on the PSC operating stability. In the operation of solar cells, thermal damage also contributes to the degradation of PSCs. Therefore, it is important to monitor *V*_OC_, particularly by operating the device, to observe the effect of thermal damage. The thermal energy from sunlight is transferred into the solar cells through (1) low energy absorption, (2) an electrical operating point, (3) encapsulation heat trapping, and (4) solar energy that is heated during charge transport. Among them, the solar energy that is heated during the charge transport can be branched into (A) thermalisation loss, (B) the junction/contact between layers, (C) radiative recombination, and (D) the thermal conductivity and resistance of materials (Figure 3a) [43,44]. These processes generate heat when the solar cells are operating. If the devices are well encapsulated, heat will be trapped, accelerating the degradation. The thermal damage to the solar cells results in a conspicuous decrease in *V*_OC_ and slight increase in *J*_SC_, with a slight decrease in FF according to the theory (Figure 3b) [45]. This implies that the device should be operated to monitor the thermal damage, reflected by the *V*_OC_ drop. Therefore, the device stability was tested by operating solar cells from this point onwards.

**Figure 4:**
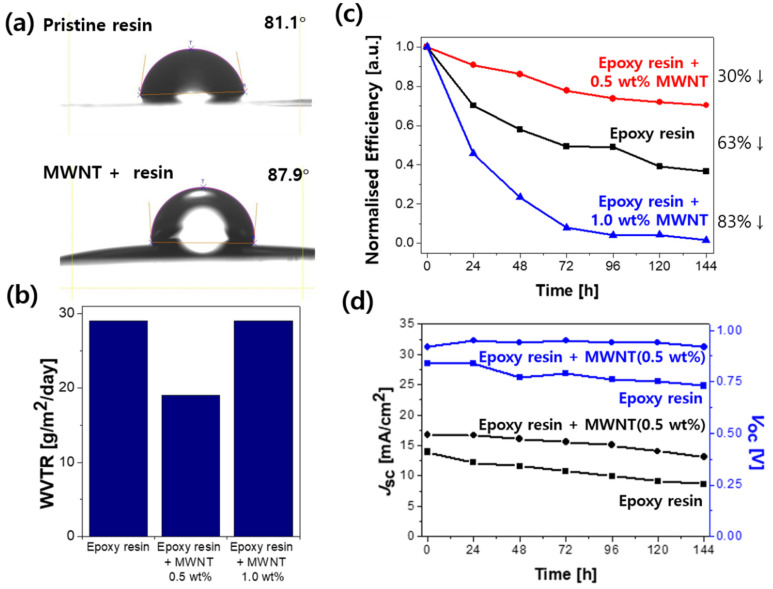
(**a**) Water contact angle of the pristine and MWNT-added UV epoxy resin; (**b**) WVTR of the MWNT-added UV epoxy resin; (**c**,**d**) Changes in (**c**) the normalised efficiency; (**d**) *J*_SC_ and *V*_OC_ of the encapsulated PSCs without (control device) and with MWNT added to the UV epoxy resin during the stability test under constant device operation.

The MWNT-added UV epoxy resin was used in the packaging process to enhance the moisture barrier property, with the encapsulating pressure of 14.71 N cm^−2^ (Figure S8). Before fabricating PSCs, we wanted to consolidate the effect of the MWNT addition and find the optimal amount of MWNT. Therefore, we conducted water contact angle tests to confirm that the addition of MWNT increased the hydrophobicity of the UV epoxy resin. The water contact angle images of epoxy resin and MWNT-added UV epoxy resin are shown in Figure 4a. The contact angle of the MWNT-added UV epoxy resin (87.9°) was larger than that of the bare epoxy resin (81.1°) [46]. For further investigation, water vapour transmittance rate (WVTR) and oxygen transmittance rate (OTR) tests were performed; they revealed the barrier ability against water and oxygen, respectively. The WVTR results showed that the 0.5 wt% of MWNT-added UV epoxy resin exhibited the lowest WVTR value of 19 g m^−2^ day^−1^, as shown in Figure 4b, Figures S9 and S10. Furthermore, the OTR test results also reveal that the optimal MWNT amount to exhibit the best barrier ability against oxygen is 0.5 wt% (Figure S11). This indicates that a specific ratio of MWNT to UV epoxy resin must be used for the good barrier property, and adding excess MWNTs can reverse the barrier effect. There are several intermolecular interactions in the carbon-based nanostructures, such as the carbon nanotubes. The main interaction of the carbon nanotubes is the π–π interaction, which represents one of the van der Waals forces [47]. However, the actual π interactions appeared between polycyclic unsaturated molecules with 10–15 carbon atoms [48]. Therefore, the carbon interactions of 0.5 wt% of MWNT-added UV epoxy resin were strong. Moreover, the amount of internal porosity of epoxy resin was decreased by the hardened carbon interaction, and the quantity of the penetrated water molecules reduced consequentially. Conversely, if the ratio of the MWNT to UV epoxy resin was higher than the specific ratio of 0.5 wt%, then the condensation of the MWNT [49] could hinder the UV light-based hardening process. The van der Waals interaction between the MWNT was stronger than the interaction between the MWNT and UV epoxy resin. Hence, the MWNT-added UV epoxy resin could be declined by UV irradiation, resulting in the accumulation of MWNTs on the surface [50]. Additionally, the permeation of the water molecules and oxygen was easier and led to the deterioration of the solar cell properties due to the decreased rigidity of epoxy resin at 1.0 wt% of the MWNT-added UV epoxy resin. Figure 4c shows that the 0.5 wt% of MWNT-added UV epoxy resin-based PSCs had a greater device stability than 1.0 wt% of MWNT-added UV epoxy resin-based PSCs and the reference PSCs without any MWNTs (Table 1). While the normalised power conversion efficiency (PCE) of the 0.5 wt% of MWNT-added UV epoxy resin-based PSCs decreased by approximately 30% over the operating time of one week, the reference devices without the MWNT addition displayed a decrease in the normalised PCE of approximately 63%. Furthermore, the 0.5 wt% of MWNT-added UV epoxy resin-based PSCs showed a high stability in all photovoltaic parameters, namely, *J*_SC_, FF, and *V*_OC_ (Figure 4d and Figures S12–S14). In the case of 0.2 wt% MWNT-added epoxy resin-based devices, the improvement in stability was not clearly visible. We ascribed this to the added amount being too small (Figure S15). The results indicated that the addition of MWNT to the UV epoxy resin increased the barrier’s ability against moisture and oxygen and released heat better. However, adding MWNTs greater than 0.5 wt% exacerbated the device stability.

## 3. Materials and Methods

### 3.1. PSC Fabrication

Patterned ITO/glass substrates with a dimension of 1.5 cm × 1.5 cm were cleaned by sonication with deionised water, acetone, and isopropyl alcohol for 10 min, respectively. After drying, the substrates were attached with heat resistant tape. A 30 μL solution measured from a mixture of 2 mg of PTAA (Sigma-Aldrich, St. Louis, MO, USA) and 500 μL toluene (Sigma-Aldrich) was dropped onto the substrate. The substrate was spin-coated at 5000 rpm for 30 s and subsequently annealed at 110 °C for 10 min in a nitrogen-filled glove box. After the coating process, the samples underwent UV–ozone treatment for 3 min to treat the hydrophobic surface of the PTAA layer and advance the adhesion property between the PTAA and FACsPbI_3_ Perovskite layer. The perovskite solution was prepared using 26 mg cesium iodide powder (CsI, Sigma-Aldrich), 154.8 mg formamidinium iodide powder (FAI, Greatcell Solar, Queanbeyan, Australia), 461 mg lead iodide (PbI_2_, Tokyo Chemical Industry, Tokyo, Japan), 74 μL dimethyl sulfoxide (DMSO), and 580 μL *N*,*N*-dimethylformamide (DMF, Sigma-Aldrich). The mixture was subsequently annealed for 40–60 min at 100 °C. A 35 μL perovskite solution was dropped onto the substrate and spin-coated at 6000 rpm for 20 s. About 10 s into the coating process, 1.75 μL of diethyl ether was sequentially dropped closer to the substrate. For the formation of the perovskite layer, the substrate was annealed for 1 min at 100 °C. The temperature was subsequently raised to 155 °C and maintained for 9 min. This was followed by spin-coating PCBM solution with a concentration of 10 mg mL^−1^ in chlorobenzene at an rpm of 3000 for 30 s. C_60_, BCP, and silver were deposited consecutively with a photomask by thermal evaporation at a speed of 5 Å s^−1^. For the encapsulation, the epoxy resin was spread on the edge of the cover glass with a dimension of 1.1 cm × 1.1 cm. The cover glass was then placed on top of the PSC in the central part of the cell. After the capping process, the cover glass was cured by UV light for 10 min. The current–voltage properties were analysed by contacting the external side of the silver electrode, which was exposed from the capped cover glass.

### 3.2. Encapsulant

The UV curable epoxy resin used to encapsulate the inverted PSC was purchased from Nagase ChemteX Corporation (XNR5516Z, Osaka Prefecture, Japan). MWNTs were purchased from CNT Co. Ltd. (Seoul, Korea). According to the technical sheet of the product, the outer diameters were 10 nm–140 nm, the tube lengths were 5–20 μm, and the purity was >99% [51,52,53].

### 3.3. Characterisations

The *J−V* curves were measured using a software-controlled source meter (Keithley 2400 Source-Meter) under dark conditions and the simulated sunlight irradiation of 1 sun (AM 1.5 G; 100 mW cm^−2^), which was generated from a solar simulator (EMS-35AAA, Ushio Spax Inc., Tokyo, Japan) with an Ushio Xe short arc lamp 500. The source meter was calibrated using a silicon diode (BS-520BK, Bunkokeiki, Tokyo, Japan). A photomask with a dimension of 3 mm × 3 mm was placed for the measurement, which defined the active area. The long-term stability test was conducted by leaving the devices in a room where the temperature was ca. 25 °C and the relative humidity was ca. 80% under constant illumination of sunlight. The external quantum efficiency (EQE) measurement system consisted of an MLS-1510 monochromator to scan the UV–Vis spectra. The cross-sectional images of the epoxy resin spread between the device, substrate, and cover glass were obtained by SEM (Hitachi High Technologies, Tokyo, Japan. S-4800). The current–voltage measurements were performed using a source meter (Agilent Technologies, Santa Clara, CA, USA. 4156C) under the illumination of simulated sunlight provided by an Oriel solar simulator equipped with an AM 1.5 G filter. The hydrophobic/hydrophilic properties of the epoxy resin and MWNT-added epoxy resin were measured using a Water contact angle analysis system. The WVTRs were measured using a Lyssy water vapour permeation analyser.

## 4. Conclusions

The inverted PSCs were fabricated, encapsulated, and monitored for their stability. A trace amount of MWNTs was added to the UV-curable epoxy resin during the packaging process to improve the barrier and heat-releasing properties of the epoxy resin, thereby protecting the devices from degradation through moisture, oxygen, and heat. Furthermore, we investigated the water and oxygen permeability and the thermal conductivity of the resin. Despite the degradation caused by moisture and oxygen being a dominant factor, the trapped heat was significant enough to accelerate the degradation. Thus, our work proposes a novel engineering approach to encapsulation technology with the potential to advance the commercialisation of electronics, especially thin-film photovoltaics.

## Figures and Tables

**Table 1:** Packaging type of the devices and solar cell performance values before and after one week of durability testing. More detailed corresponding *J*–*V* curves, EQE, and statistical information can be found in Figures S7, S15 and S16).

Device Type\Device Performance		*J*_SC_ [mA/cm^2^]	*V*_OC_ [V]	FF	PCE [%]
Feature	Stability Time				
Before encapsulation	N/A	19.4	0.94	64.8	11.8
Encapsulation by 4.90 N cm^−2^ pressure	Initial	11.3	0.97	28.67	3.15
	one week	2.31	0.96	15.33	0.34
Encapsulation by 14.71 N cm^−2^ pressure	Initial	13.9	0.99	28.69	3.96
	one week	8.63	0.85	26.72	1.96
Encapsulation with MWNT (0.5 wt%)-added epoxy resin	Initial	16.7	0.92	61.90	9.51
	one week	13.1	0.92	55.51	6.69
Encapsulation with MWNT (1.0 wt%)-added epoxy resin	Initial	16.8	0.99	39.08	6.50
	one week	1.19	0.87	10.62	0.11

## Data Availability

Not applicable.

## Supplementary Materials

The following are available online, 1. Light Absorbance Stability of Devices; 2. Encapsulation Approaches; 3. Stability of Devices with Different Passivation Structures; 4. Devices with Different Structures; 5. Devices with Different Applied Pressures; 6. Performance of Unencapsulated Devices; 7. Images of MWNT added UV-epoxy resin; 8. WVTR Data; 9. WVTR Sample Preparation; 10. OTR Data; 11. *J*_SC_ and *V*_OC_ during Stability Test; 12. Performance of 0.5 wt% MWNT-added Encapsulated Devices; and 13. Stability of 0.2 wt% MWNT-added Encapsulated Devices.

## Sample Availability

Samples of the compounds are not available.
