# Supplementary material for: Multi-Walled Carbon Nanotube-Assisted Encapsulation Approach for Stable Perovskite Solar Cells
Source: Molecules. 2021 Aug 20;26(16):5060. doi: 10.3390/molecules26165060 (PMC8399998; doi:10.3390/molecules26165060)
Supplement: Supplementary file 1 [file molecules-26-05060-s001.zip › molecules-1321452-supplementary.pdf]

Supporting Information

Multi-Walled Carbon Nanotube-assisted Encapsulation Approach for Stable Perovskite Solar Cells

Jin-Myung Choi[1,2], Hiroki Suko[3], Kyusun Kim[1], Jiye Han[1,2], Sangsu Lee[1,2], Yutaka Matsuo[3,4], Shigeo Maruyama[3], Il Jeon[1,2]*, Hirofumi Daiguji[3]*

[1]Department of Chemistry Education, Graduate School of Chemical Materials, Crystal Bank Institute, Pusan National University, Busan 46241, South Korea

[2]Department of Nano Fusion Technology, Pusan National University, Busan 46241, Republic of Korea

[3]Department of Mechanical Engineering, School of Engineering, The University of Tokyo, Tokyo 113-8656, Japan

[4]Department of Chemical System Engineering, Graduate School of Engineering, Nagoya University, Nagoya 464-8603, Japan

* Corresponding author, E-mail address: il.jeon@spc.oxon.org (Prof. I. Jeon), daiguji@thml.t.u-tokyo.ac.jp (Prof. H. Daiguji)

<u>Contents</u>

1. Light Absorbance Stability of Devices

2. Encapsulation Approaches

3. Stability of Devices with Different Passivation Structures

4. Devices with Different Structures

5. Devices with Different Applied Pressures

6. Performance of Unencapsulated Devices

7. Images of MWNT added UV-epoxy resin

8. WVTR Data

9. WVTR Sample Preparation

10. OTR Data

11. J_{SC} and V_{OC} during Stability Test

12. Performance of 0.5 wt% MWNT-added Encapsulated Devices

13. Stability of 0.2 wt% MWNT-added Encapsulated Devices

1. Light Absorbance Stability of Devices

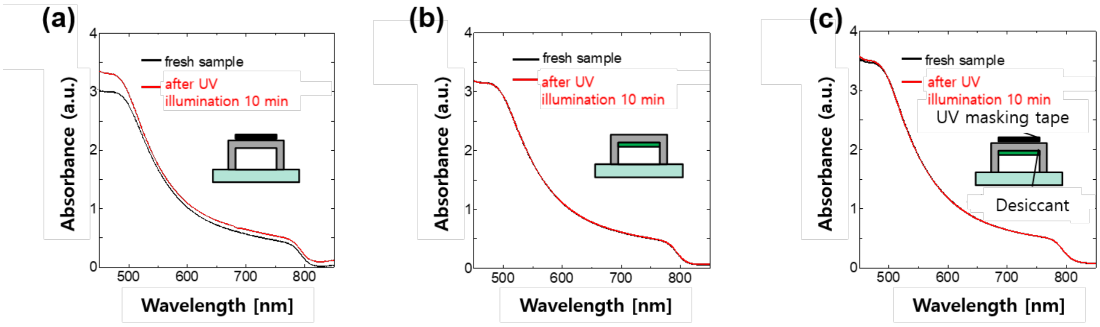

Figure S1. (**a–c**) Light absorbance of the perovskite film before and after UV illumination for 10 minutes in three different encapsulation structures: (**a**) with a UV masking, (**b**) with a desiccant (getter), and (**c**) with both a UV masking and a desiccant.

2. Encapsulation Approaches

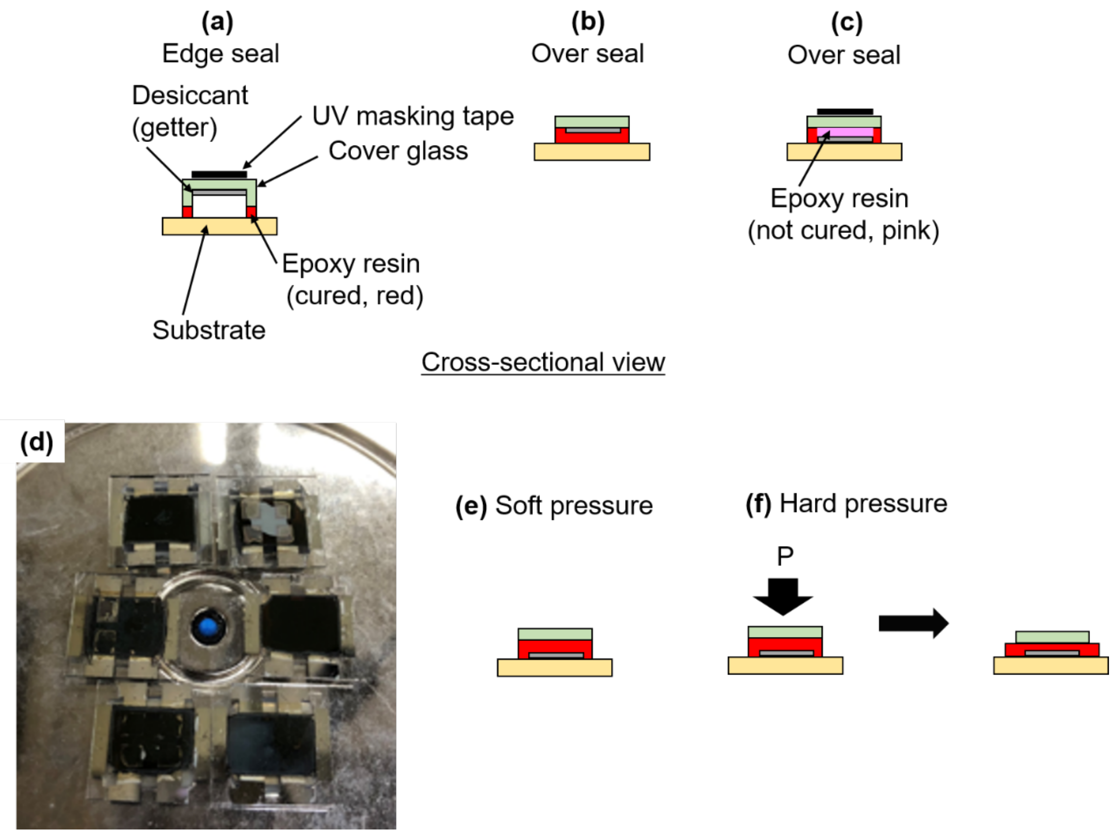

Figure S2. (**a**–**c**) Three different encapsulation structures: (**a**) edge sealing with both a desiccant and a UV masking tape, (**b**) over sealing with only a desiccant, and (**c**) over sealing with both a desiccant and a UV masking tape, which leaves the centre of epoxy resin not cured; (**d**) Photographic image of the devices made by the suggested structures; (**e**–**f**) Two different pressures applied during the packaging.

3. Stability of Devices with Different Passivation Structures

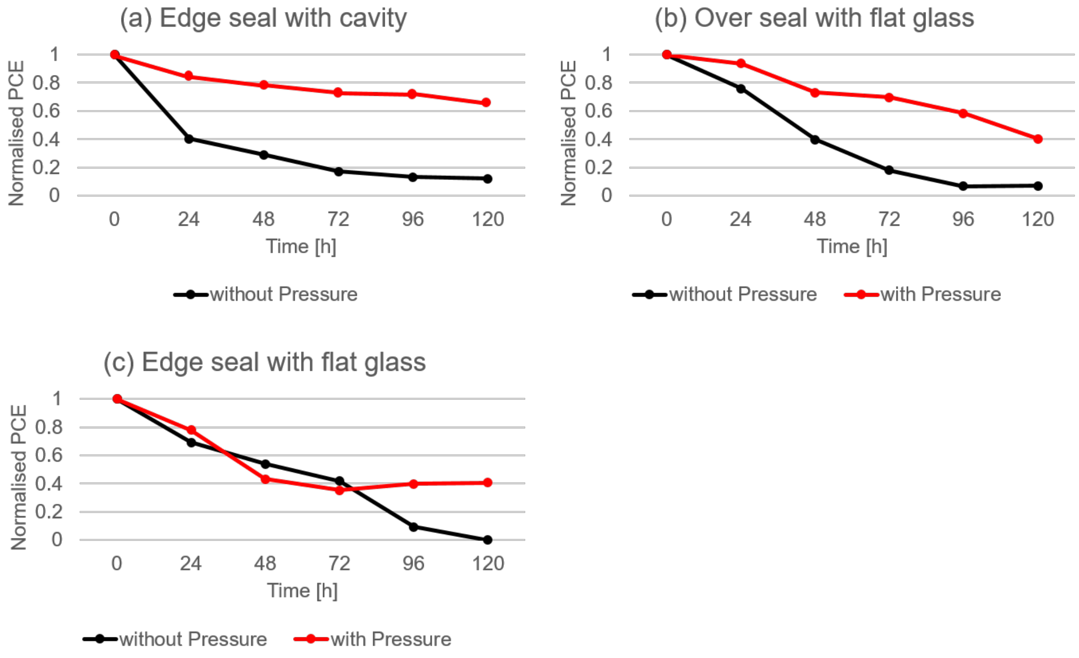

Figure S3. Normalised power conversion efficiency of the (**a**) edge seal with cavity; (**b**) over seal with flat glass; and (**c**) edge seal with flat glass during 120 min stability test.

4. Devices with Different Structures

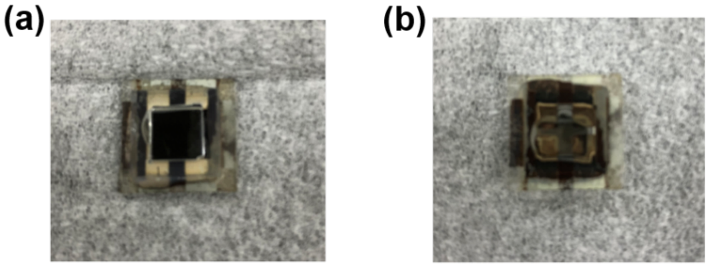

Figure S4. Photographic images of (**a**) edge seal- and (**b**) over seal-encapsulated samples.

5. Devices with Different Applied Pressures

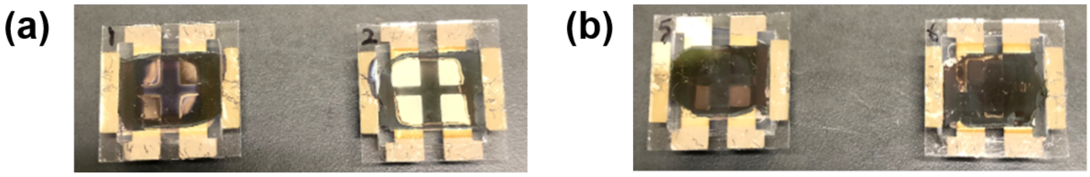

Figure S5. Images before and after one week of durability testing: (**a**) 4.90 N cm^{-2} pressure- and (**b**) 14.71 N cm^{-2} pressure-encapsulated perovskite solar cells**.**

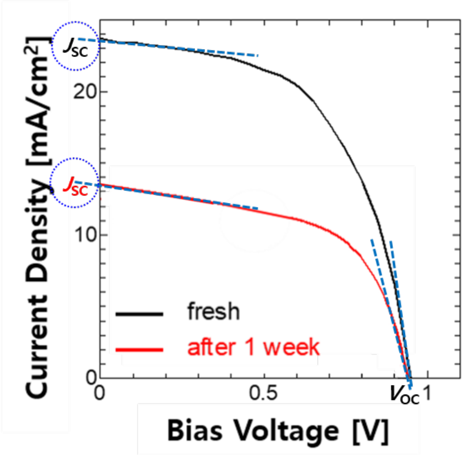

Figure S6. *J*–*V* curves of the encapsulated PSCs under 14.71 N cm^{-2} with a nitrogen gun blown constantly during the stability test to keep the device temperature low.

6. Performance of Unencapsulated Devices

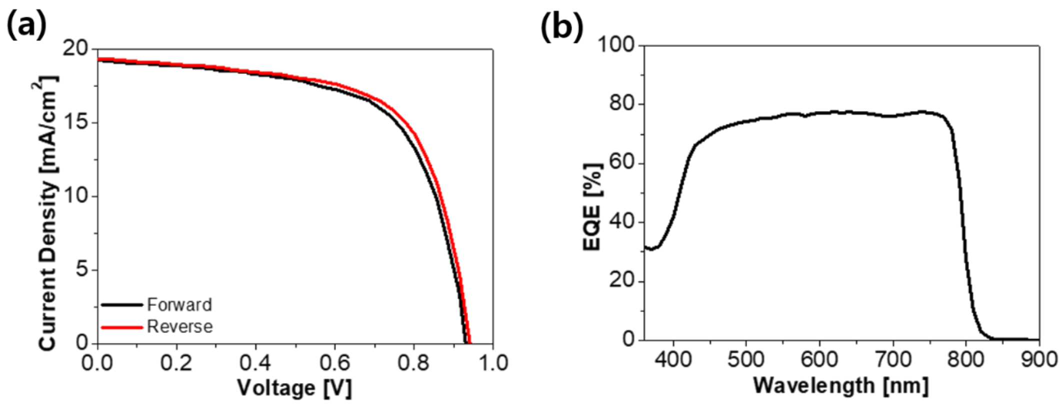

Bias	J_{SC} [mA/cm^2]	V_{OC} [V]	FF	PCE [%]
Forward	19.26	0.93	63.76	11.46
Reverse	19.36	0.94	64.83	11.84

Figure S7. (**a**) Forward and reverse bias *J*–*V* curves, (**b**) EQE, and photovoltaic table of the unencapsulated PSCs,

7. Images of MWNT-added UV-epoxy resin

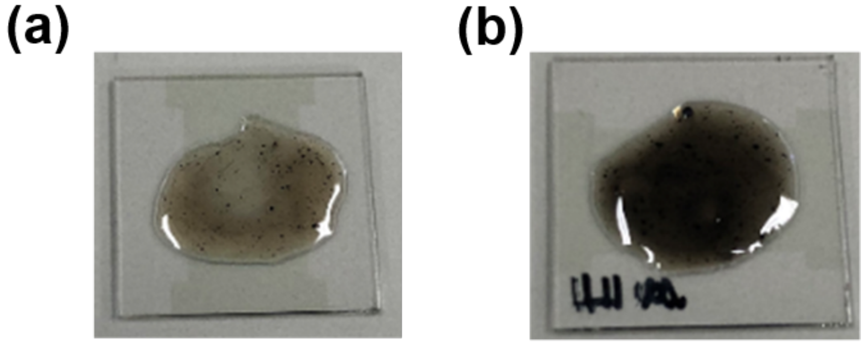

Figure S8. Images of (**a**) 0.5-wt%; and (**b**) 1.5-wt% multi-walled nanotube (MWNT)-added UV epoxy resin.

8. Water vapour transmittance rate (WVTR) Data

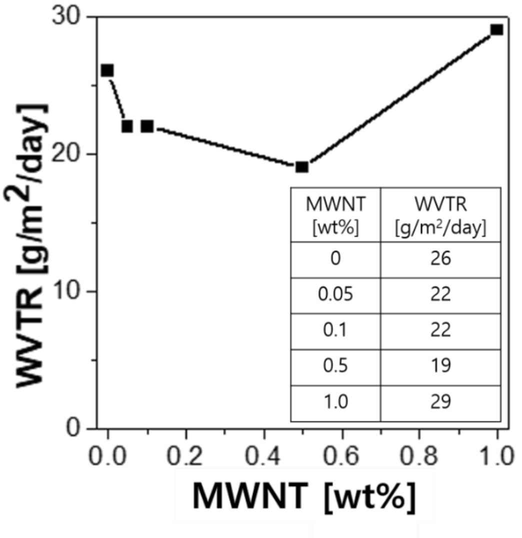

Figure S9. Water vapour transmittance rate (WVTR) of the MWNT-added UV epoxy resin with respect to the wt% of the MWNT.

9. WVTR Sample Preparation

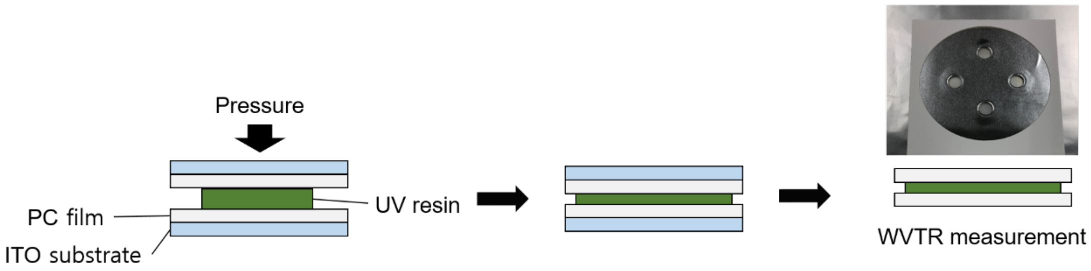

Figure S10. Schematic of the sample preparation for the WVTR measurement.

10. Oxygen transmittance rate (OTR) Data

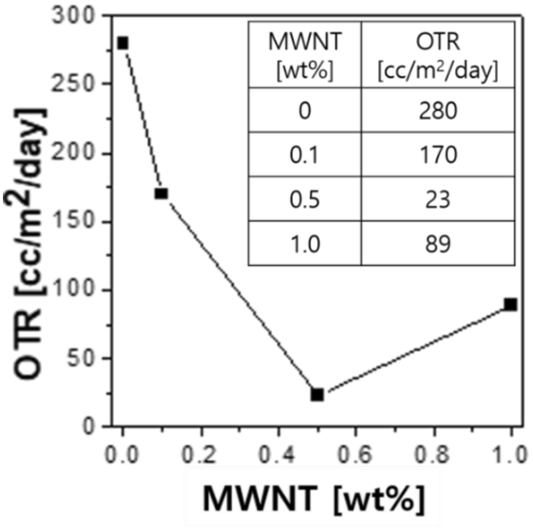

Figure S11. Oxygen transmittance rate (OTR) of the MWNT-added UV epoxy resin with respect to the wt% of the MWNT.

11. J_{SC} and V_{OC} during Stability Test

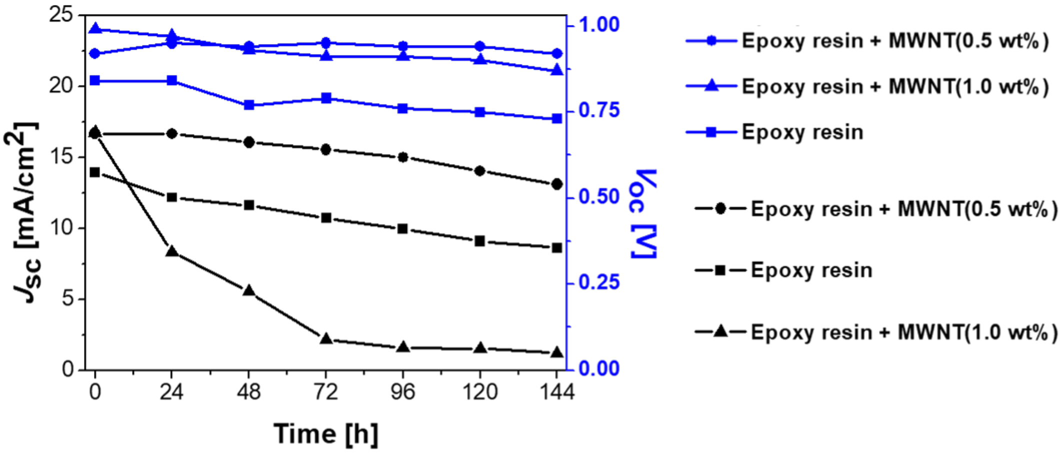

Figure S12. J_{SC} and V_{OC} of the 0, 0.5, and 1.0 wt% of MWNT-added UV epoxy resin.

12. Performance of 0.5 wt% MWNT-added Encapsulated Devices

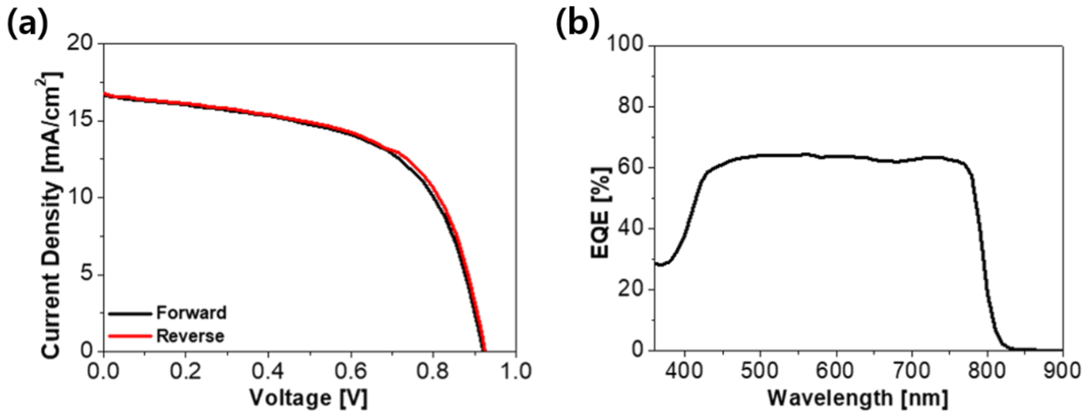

Bias	J_{SC} [mA/cm^2]	V_{OC} [V]	FF	PCE [%]
Forward	16.6	0.91	59.1	8.93
Reverse	16.7	0.92	60.1	9.24

Figure S13. (**a**) Forward and reverse bias *J*–*V* curves, (**b**) EQE, and photovoltaic table of the 0.5 wt% MWNT-added encapsulated PSCs.

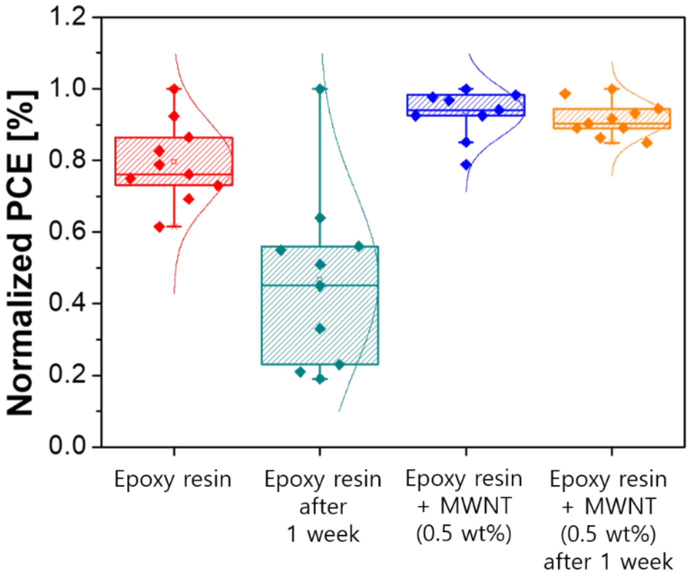

Figure S14. Statistical analysis of the 0.5 wt% MWNT-added encapsulated PSCs before and after the stability test.

13. Stability of 0.2 wt% MWNT-added Encapsulated Devices

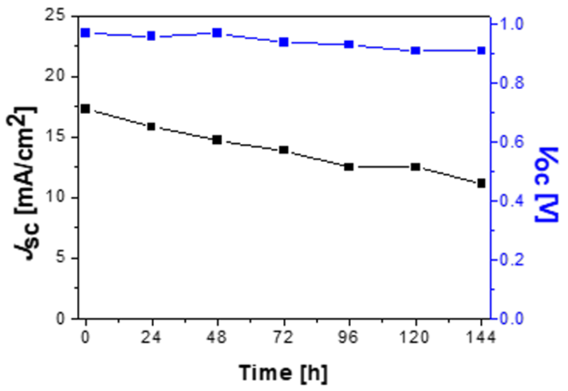

Figure S15. Stability of the 0.2 wt% MWNT-added Encapsulated Devices.
